# Sacubitril/Valsartan vs ACE Inhibitors or ARBs: A Systematic Review and Meta-Analysis of Randomized Trials

**DOI:** 10.1016/j.jacadv.2025.101598

**Published:** 2025-02-18

**Authors:** Endurance Evbayekha, Abiodun Benjamin Idowu, Shane LaRue

**Affiliations:** a St. Luke's Hospital, Chesterfield, Missouri, USA; b Einstein Medical Center, Philadelphia, Pennsylvania, USA

**Keywords:** angiotensin-converting enzyme inhibitor, angiotensin receptor blocker, heart failure, HFmrEF, HFpEF, HFrEF, neprylisin inhibitor, sacubitril/valsartan

## Abstract

**Background:**

Sacubitril/valsartan (SAV) is crucial for managing heart failure (HF). Randomized clinical trials have shown SAV's superiority over angiotensin-converting enzyme inhibitors (ACEI) and angiotensin receptor blockers (ARBs) in reducing N-terminal pro-B-type natriuretic peptide levels. However, results for cardiovascular (CV) mortality, HF rehospitalization, and all-cause mortality have been mixed.

**Objectives:**

This study aimed to determine hard endpoints among the population with HF treated with SAV vs ACEI/ARBs and conduct a comprehensive risk-benefit analysis of the safety profile for SAV vs ACEI/ARB.

**Methods:**

We queried PubMed, EMBASE, Scopus, and the Cochrane Central Register of Controlled Trials for randomized clinical trials from inception to November 2023. We included studies that compared SAV to ACEI or ARBs and reported hard endpoints, including all-cause mortality, CV mortality, and HF rehospitalizations. Random effect model was used, and categorical values were analyzed using risk ratios (RRs) and 95% CI. The I^2^ test was used to assess between-study heterogeneity. Publication bias was assessed via funnel plots and the Egger test. This study was registered in PROSPERO (CRD42024497661).

**Results:**

The study included a total of 14 trials (n = 25,167). SAV reduced all-cause mortality in the population with an ejection fraction (EF) ≤40% (RR: 0.88; 95% CI: 0.81-0.94; *P* = 0.0006), but not in those with EF >40% (RR: 0.97; 95% 0.85-1.11; *P* = 0.67). There was no difference in CV mortality across EF spectrums (RR: 0.9; 95% CI: 0.79-1.03; *P* = 0.13). HF readmission was lower in the SAV-treated group regardless of EF (RR: 0.85; 95% CI: 0.79-0.91; *P* = 0.00001).

**Conclusions:**

The SAV-treated group, across all EF spectrum, was less likely to be rehospitalized than the ACEI/ARB-treated group. However, all-cause mortality reduction was only noted in the SAV group with EF <40%. No reduction in CV-related mortality was observed across the EF spectrum.

Heart failure (HF) is classified into 3 groups based on left ventricular ejection fraction (LVEF) per the current guidelines: HF with reduced EF (HFrEF); EF <40%, HF with preserved EF; EF ≥50%, and HF with mildly reduced EF; EF 41% to 49%.^1^ HF with mildly reduced EF is a topic of much debate, with some suggesting that HFrEF and HF with preserved EF should coincide at an EF of 45%.^2^ Nonetheless, these cutoffs were established because individuals with a lower EF demonstrate a higher risk of HF-related hospitalization and mortality.^1^

It has been nearly 35 years since angiotensin-converting enzyme inhibitors (ACEI) became part of the standard regimen for HF. Enalapril reduced mortality risk in the SOLVD (Studies of Left Ventricular Dysfunction) and CONSENSUS (Cooperative North Scandinavian Enalapril Survival Study) trials.^3^^,^^4^ However, in 2014, the PARADIGM-HF (Prospective Comparison of angiotensin receptor-neprilysin inhibitor (ARNI) with ACEI to Determine Impact on Global Mortality and Morbidity in Heart Failure) study demonstrated that ARNI, specifically sacubitril/valsartan (SAV), was superior to ACEI over multiple important endpoints, including reducing cardiovascular (CV) death and HF hospitalizations.^5^ Neprilysin, a neutral endopeptidase, is vital in breaking down endogenous vasoactive peptides like natriuretic peptides, bradykinin, and adrenomedullin. An elevation in the concentrations of these substances due to neprilysin inhibition counters the overactivation of neurohormones responsible for vasoconstriction, maladaptive remodeling, and sodium retention.^5^ Therefore, neprilysin, in addition to angiotensin II antagonism, may demonstrate superiority over ACEI/angiotensin receptor blockers (ARBs) alone and improve HF outcomes.

The current body of knowledge presents a problem in evaluating SAV in HF management. Studies focusing on “hard” endpoints, such as CV-related mortality, HF rehospitalization, and all-cause mortality, have yielded conflicting and predominantly negative results.^6^^,^^7^ These hard endpoints are crucial as they directly measure HF progression and the impact of treatment on patient survival and hospitalization rates.^8^

Conversely, recent studies have shifted focus toward “soft” endpoints, including N-terminal pro-B-type natriuretic peptide and 6-minute walking distance.^9^^,^^10^ While measuring and demonstrating changes in a shorter time frame is easier, these surrogate endpoints, although important and strong prognosticator tools, do not accurately translate to improved survival or decreased hospitalization.^9^^,^^10^ This study aimed to determine hard endpoints among the population with HF treated with SAV vs ACEI/ARBs as our primary outcome. Additionally, as a secondary outcome, we examined the safety profile of SAV vs ACEI/ARB to conduct a comprehensive risk-benefit analysis, which is crucial for informing clinicians' decisions regarding medication prescription. We hypothesized that SAV may lack consistency in attaining favorable endpoints across the EF spectrum.

## Methods

This study was conducted according to the Preferred Reporting Items for Systematic Review and Meta-Analysis guidelines.^11^ The study methodology was registered and approved by the International Prospective Register of Systematic Reviews, PROSPERO (CRD42024497661).

### Search strategy

We queried PubMed, EMBASE, Scopus, and Cochrane Central Register of Controlled Trials from inception to November 2023 for randomized controlled trials that compared SAV to ACEI or ARBs and reported at least one of our prespecified primary outcomes. For secondary outcomes, we performed a subanalysis of the selected studies that reported the efficacy and safety of SAV in patients with HF. Searched keywords including “heart failure,” “systolic dysfunction,” “heart failure with reduced ejection fraction,” “diastolic heart failure,” “heart failure with preserved ejection fraction,” “ARNI,” “Entresto,” “Sacubitril/Valsartan,” “LCZ696,” “Angiotensin-converting enzyme inhibitor,” “ACEI,” “Angiotensin receptor blocker,” “ARB,” were combined using appropriate Boolean operators “AND” and “OR.” We did not apply language, publication year, or country filters.

### Study review and selection criteria

Search results were exported into Covidence, a web-based collaboration systematic review software (Veritas Health Innovation). Two reviewers (E.E. and A.I.) independently screened the titles and abstracts of the shortlisted articles to identify potentially eligible studies. The same 2 investigators later read the full texts of retained relevant articles. A third investigator blindly resolved any inclusion-related discrepancy. The bibliographies of eligible articles were also examined to identify additional studies. The prespecified inclusion criteria were: 1) randomized controlled trial or clinical trials; 2) human studies; 3) 2-arm parallel groups with a control group (taking either ACEI or ARB) and an intervention arm (taking SAV); 4) study must report at least one endpoint of interest.

### Endpoint of interest

Priori-defined outcomes of interest include efficacy endpoint (all-cause mortality, CV-related mortality, and HF rehospitalization) and safety outcomes. Safety events of interest included drug discontinuation due to adverse effects, worsening renal function, hyperkalemia, and incidences of angioedema as reported per each study at their longest follow-up. Subgroup sensitivity analysis based on LVEF was conducted for efficacy endpoints.

### Data extraction and statistical analysis

Two independent reviewers (E.E. and A.I.) extracted data using a predefined Microsoft Excel sheet. Collated data included study details (author, year of publication, number of participants, and follow-up duration), patient characteristics (age, gender, race/ethnicity, comorbidities, NYHA functional class, EF, and baseline medications), and outcomes. The risk of bias was assessed using the Revised Cochrane Risk-of-Bias Tool for Randomized Trials tool. Publication bias was assessed visually using the funnel plot diagram and Egger test.^12^ A frequency meta-analysis was done for quantitative data. An inverse-variance weighting and random effects model meta-analysis was performed for the included studies in line with the DerSimonian and Laird method in anticipation of the heterogeneity of the eligible studies.^13^ Higgins's I^2^ test was used to determine between-study heterogeneity, with an I^2^ of 0% to 29%, 30% to 49%, and >50% signifying negligible, moderate, and substantial inconsistencies, respectively.^14^^,^^15^ Effect estimate was reported as risk ratio (RR) as 9 of the 14 included trials had no time-to-event analysis for outcomes of interest. In the absence of hazard ratio reporting in most studies, similar to prior published study-level frequentist meta-analyses, we calculated RR from the reported frequency of events.^16^ All statistical tests were conducted at a 2-sided 5% significance level using Review Manager 5.4 (Cochrane Collaboration) and STATA, version 18.

## Results

### Study and patient characteristics

**Figure 1:**
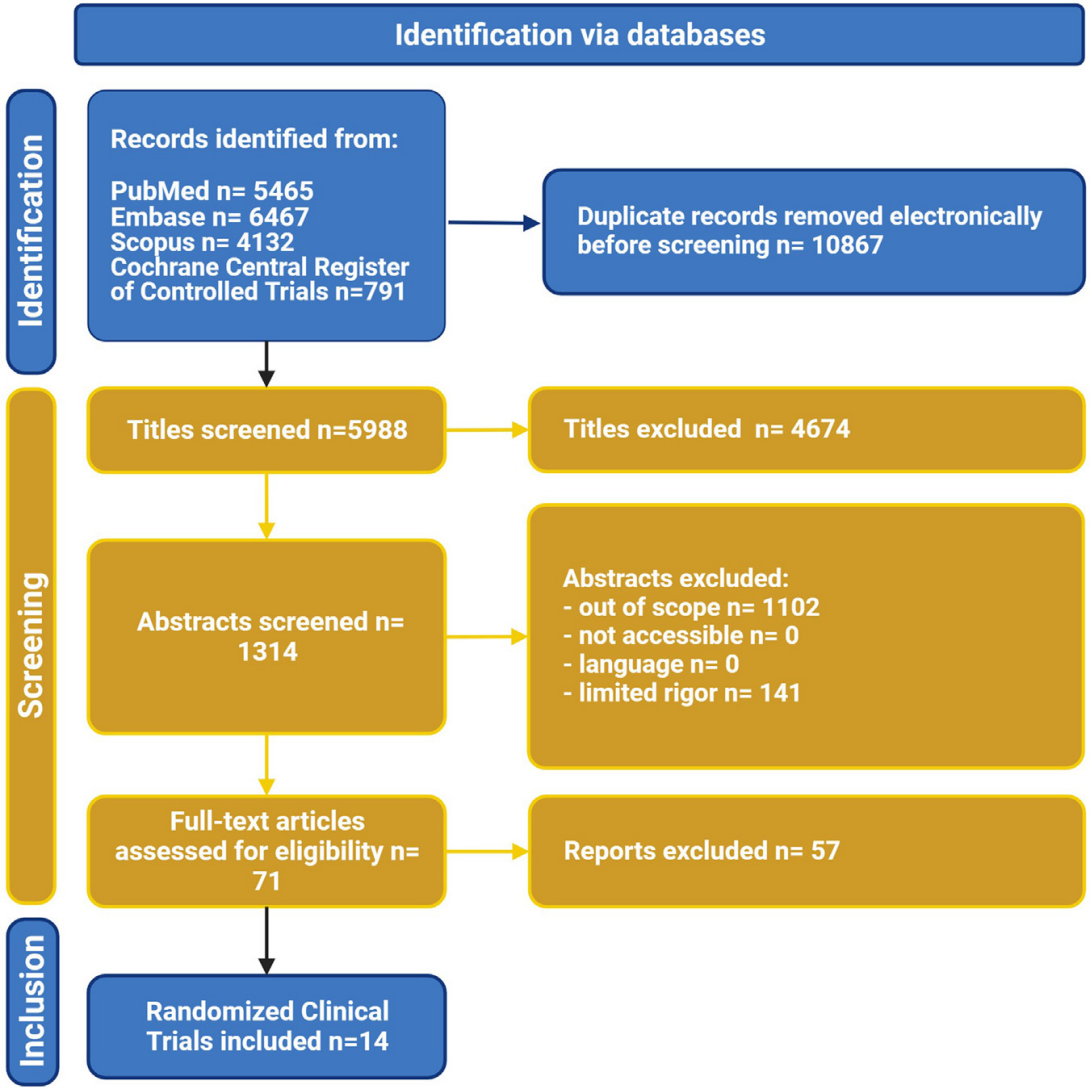
Preferred Reporting Items for Systematic Reviews and Meta-Analyses Flowchart Demonstrates Systematic Query of Various Databases

**Figure 2:**
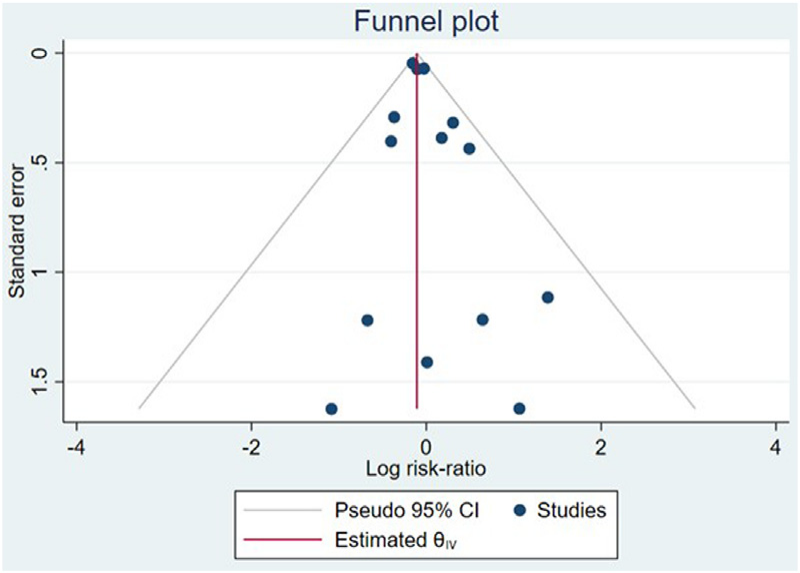
Funnel Plot Diagram

**Figure 3:**
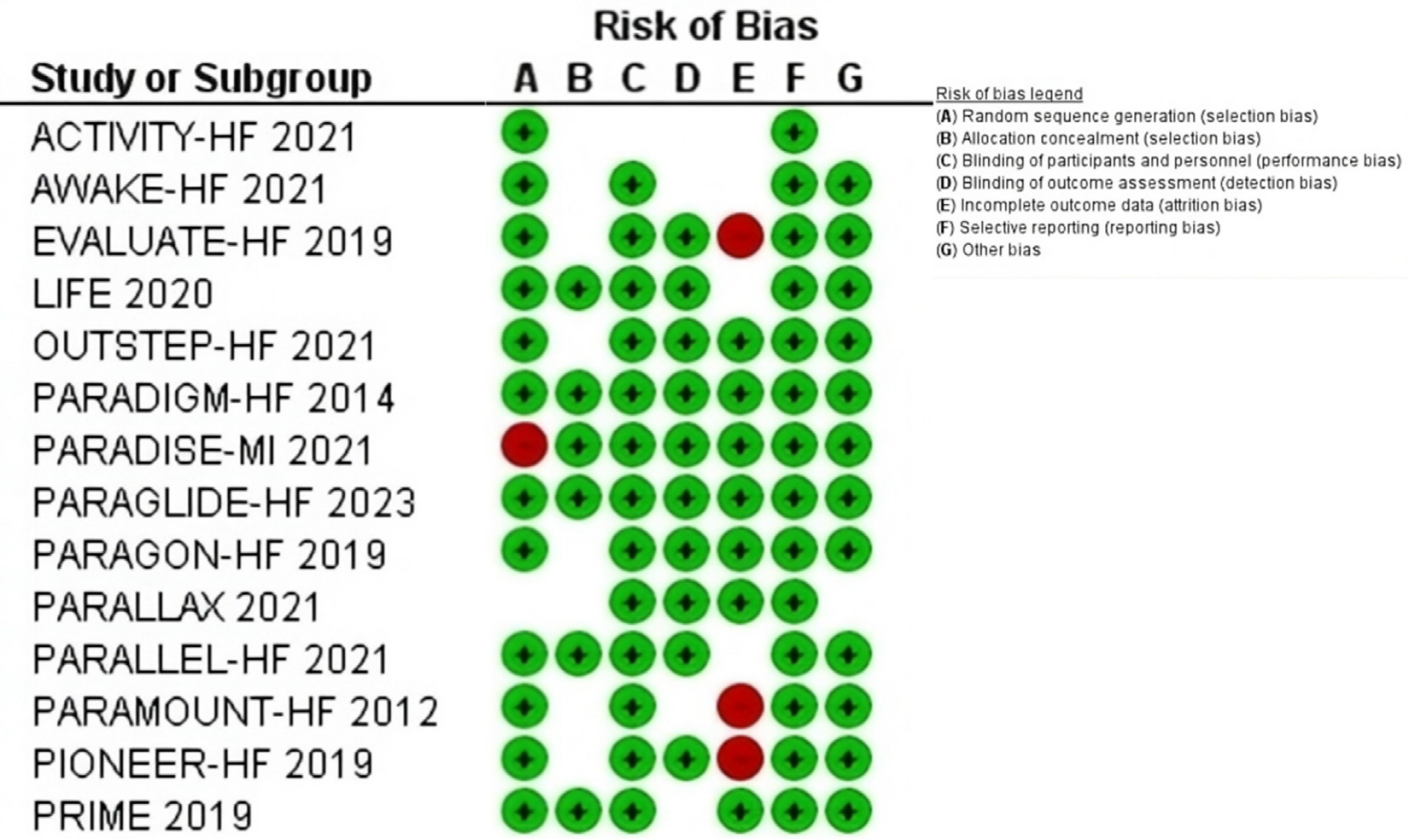
Overall Risk of Bias of Included Studies Interpretation: low risk color-coded green, blank represents unclear risk, red indicates high risk.

The systematic search identified a total of 5,988 articles after the exclusion of duplicates (Figure 1). After screening and applying prespecified inclusion criteria, 14 randomized clinical trials with 25,167 patients (31.7% were female, 58.6%, and 23.6% were in NYHA functional class II and III, respectively) (see Table 1). The included studies have no significant risk of publication bias (Egger's test for a regression intercept gave a *P* value of 0.225) (Figure 2) and an overall low risk of bias (see Figure 3).

**Table 1:** Baseline Characteristics of Each Randomized Clinical Trial

Trial	First Author	Year	Mean Age, y	Female (%)	Target Intervention Dose	Target Control Dose	Population	Follow-Up Duration (Mean/Median^a^)	Primary Outcome
PARAMOUNT-HF	Solomon et al^17^	2012	71	56.5	Sacubitril/Valsartan 200 mg twice daily	Valsartan 160 mg twice daily	NYHA class II, EF >50%	12 wk	Reduction in NT-proBNP levels with SAV compared to valsartan
PARADIGM-HF	McMurray et al^5^	2014	64	21	Sacubitril/Valsartan 200 mg twice daily	Enalapril 10 mg twice daily	NYHA II-IV, EF ≤40%	27 mo	Greater composite outcome of CV death and HF admissions (HR: 0.80; 95% CI: 0.73-0.87; *P* < 0.001)
PRIME	Kang et al^18^	2018	62.6	39	Sacubitril/Valsartan 97/103 mg twice daily	Valsartan 160 mg twice daily	NYHA class II-III, EF 25%-<50%	12 mo	Change in effective regurgitant orifice area of functional MR
PIONEER-HF	Velazquez et al^19^	2019	61	27.9	Sacubitril/Valsartan 97/103 mg twice daily	Enalapril 10 mg twice daily	Hospitalized for ADHF, EF ≤40%	8 wk	Greater time-averaged reduction in NT-proBNP in SAV group (HR: 0.71; 95% CI: 0.63-0.81; *P* < 0.001)
PARAGON-HF	Solomon et al^7^	2019	72.7	51.7	Sacubitril/Valsartan 97/103 mg twice daily	Valsartan 160 mg twice daily	NYHA II-IV, EF ≥45%	35^a^	No between-group difference in composite of CV death and total HHF (rate ratio: 0.87; 95% CI: 0.75-1.01)
EVALUATE-HF	Desai et al^20^	2019	67.3	23.5	Sacubitril/Valsartan 97/103 mg twice daily	Enalapril 10 mg twice daily	NYHA class I-III, EF <40%	12 wk	Change from baseline to week 12 in aortic characteristic impedance
AWAKE-HF	Khandwalla et al^21^	2020	63	22.9	Sacubitril/Valsartan 97/103 mg twice daily	Enalapril 20 mg daily	NYHA II-III, EF <40%	8 wk	No between-group difference in device measured change in mean activity counts (GMR: 0.9456; *P* = 0.0895)
OUTSTEP-HF	Piepoli et al^9^	2020	66.9	21.3	Sacubitril/Valsartan 200 mg twice daily	Enalapril 10 mg twice daily	NYHA II-IV, EF ≤40%	12 wk	No between-group difference in change from baseline in 6MWD (least squares mean difference: 8.98 m)
PARALLEL-HF	Tsutsui et al^22^	2021	67.8	13.9	Sacubitril/Valsartan 200 mg twice daily	Enalapril 10 mg twice daily	NYHA II-IV, EF ≤35%	33.9 mo^a^	No between-group difference in composite of CV death and HHF (HR: 1.09; 95% CI: 0.65-1.82; *P* = 0.6260)
PARALLAX	Pieske et al^10^	2021	72.6	50.7	Sacubitril/Valsartan 97/103 mg twice daily	Enalapril 10 mg twice daily or valsartan 160 mg twice daily	NYHA II-IV, EF ≥40%	24 wk	Greater reduction in NT-proBNP in SAV group (GMR: 0.84; *P* < 0.001); No difference in 6MWD (*P* = 0.42)
PARADISE-MI	Pfeffer et al^6^	2021	63.7	24.1	Sacubitril/Valsartan 97/103 mg twice daily	Ramipril 5 mg twice daily	AMI, EF ≤40%	22 mo^a^	No between-group difference in composite of CV death or incident HF (HR: 0.90; *P* = 0.17)
ACTIVITY-HF	Halle et al^23^	2021	66.9	19	Sacubitril/Valsartan 97/103 mg twice daily	Enalapril 10 mg twice daily	NYHA III, EF <40%	12 wk	No between-group difference in peak VO_2_ (least squares mean difference: 0.32 mL/min/kg; *P* = 0.2327)
LIFE	Mann et al^24^	2021	59.3	27	Sacubitril/Valsartan 200 mg twice daily	Valsartan 160 mg twice daily	NYHA IV, EF ≤35%	24 wk	No between-group difference in NT-proBNP change from baseline (AUC ratio 0.95; *P* = 0.45)
PARAGLIDE-HF	Mentz et al^25^	2023	70	52	Sacubitril/Valsartan 97/103 mg twice daily	Valsartan 160 mg twice daily	Within 30 d of WHF event, EF ≥40%	8 wk	Greater reduction in NT-proBNP in SAV group (ratio of change 0.85; *P* = 0.049)

6MWD = 6-minute walking distance; ACTIVITY-HF = Assessment of Treatment With Sacubitril/Valsartan on Cardiac and Renal Function in Heart Failure Patients; ADHF = acute decompensated heart failure; AMI = acute myocardial infarction; AUC = area under the curve; CV = cardiovascular; EF = ejection fraction; EVALUATE-HF = Effect of Sacubitril/Valsartan Versus Enalapril on Aortic Stiffness in Heart Failure With Reduced Ejection Fraction; GMR = geometric mean ratio; HF = heart failure; HHF = heart failure hospitalization; LIFE = Comparison of Sacubitril/Valsartan Versus Valsartan Alone in Advanced Heart Failure; MR = mitral regurgitation; NT-proBNP = N-terminal pro-B-type natriuretic peptide; NYHA = New York Heart Association; OUTSTEP-HF = randOmized stUdy using acceleromeTry to compare Sacubitril/valsarTan and Enalapril in Patients with Heart Failure With Reduced Ejection Fraction; PARADIGM-HF = Prospective Comparison of angiotensin receptor-neprilysin inhibitor (ARNI) with ACEI to Determine Impact on Global Mortality and Morbidity in Heart Failure; PARAGLIDE-HF = Prospective comparison of ARNI with ARB Given following stabilization In DEcompensated HFpEF; PARALLAX = Prospective Comparison of ARNI vs Comorbidity-Associated Conventional Therapy on Quality of Life and Exercise Capacity; PARALLEL-HF = Prospective comparison of ARNI with ACEI to determine the novel beneficial treatment value in Japanese Heart Failure patients; PARAMOUNT-HF = Prospective comparison of ARNI with ARB on Management of Heart Failure with Preserved Ejection Fraction; PIONEER-HF = Comparison of Sacubitril/Valsartan Versus Enalapril on Effect on NT-proBNP in Patients Stabilized From an Acute Heart Failure Episode; PRIME = Pharmacological Reduction of Functional, Ischemic Mitral Regurgitation; SAV = sacubitril/valsartan; VO_2_ = volume of oxygen consumed per minute; WHF = worsening heart failure.

a Median follow up duration.

### Efficacy endpoints meta-analysis

#### All-cause mortality

**Figure 4:**
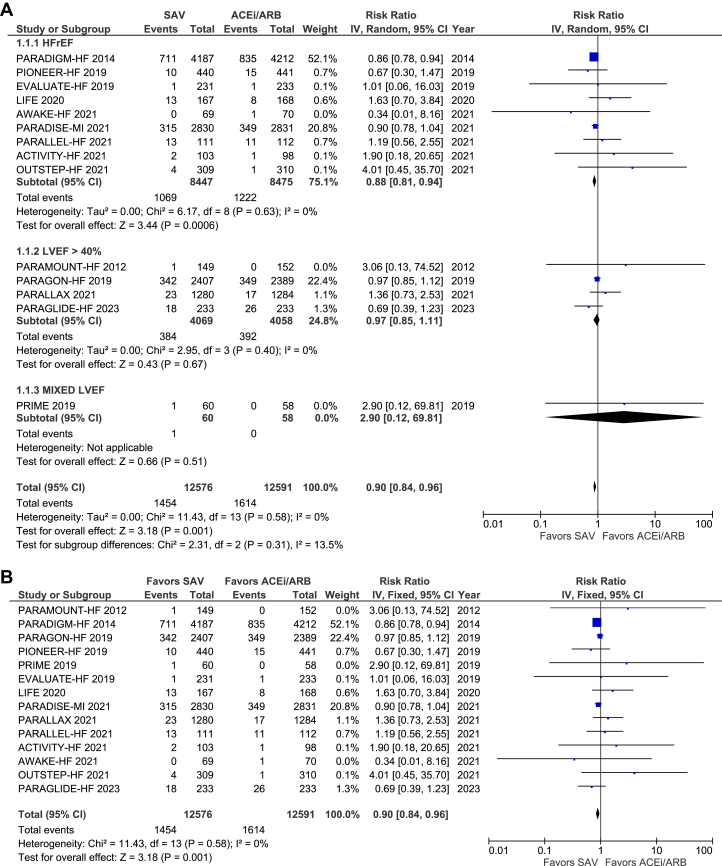
Forest Plots Showing the Effect of SAV in Comparison to ACEi/ARB on All-Cause Mortality and All-Cause Mortality Using Fixed-Effects Meta-Analysis (A) Forest plots showing the effect of SAV in comparison to ACEI/ARB on all-cause mortality. (B) Forest plots showing the effect of SAV compared to ACEI/ARB on all-cause mortality using fixed effect meta-analysis. ACEI = angiotensin-converting enzyme inhibitor; ARB = angiotensin receptor blocker; ARNI = angiotensin receptor-neprilysin inhibitor; HFrEF = heart failure with reduced ejection fraction; LVEF = left ventricular ejection fraction; SAV = sacubitril/valsartan.

SAV was significantly superior to either ACEI or ARB alone in reducing all-cause mortality across the combined population with HF (RR: 0.90; 95% CI: 0.84-0.96; *P* = 0.001). However, the subgroup analysis showed a reduction in all-cause mortality only in the population with EF ≤40% (RR: 0.88; 95% CI: 0.81-0.94; *P* = 0.0006). In those with EF >40%, all-cause mortality was not significantly different between SAV-treated and ACEI/ARB-treated groups (RR: 0.97; 95% 0.85-1.11; *P* = 0.67) (Figure 4). We utilized the random effects method in Figure 4 because the random effect model gives identical results to the fixed effect method when no heterogeneity exists among the studies.^26^^(p10)^

#### Cardiovascular death

**Figure 5:**
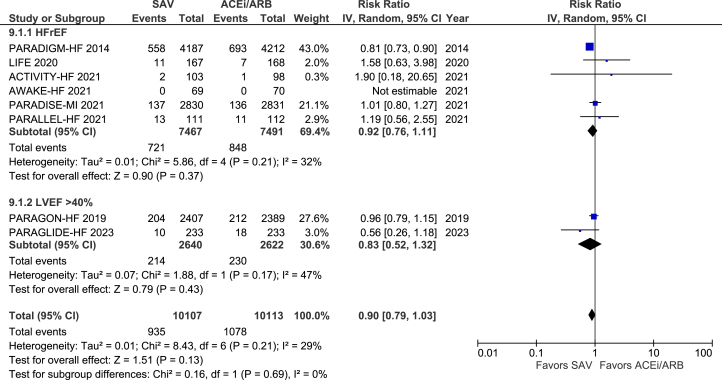
Cardiovascular-Related Death Abbreviations as in Figure 4.

There was no statistically significant difference in cardiac-related death between the SAV cohort and non-SAV group (RR: 0.9; 95% CI: 0.79-1.03; *P* = 0.13). The nonsuperiority of SAV vs ACEI/ARB in relation to CV-related death persisted irrespective of the patient's baseline EF-EF <40% (RR: 0.92; 95% CI: 0.76-1.11; *P* = 0.37) and EF >40% (RR: 0.83; 95% CI: 0.52-1.32; *P* = 0.43) (Figure 5).

#### Heart failure readmission

**Figure 6:**
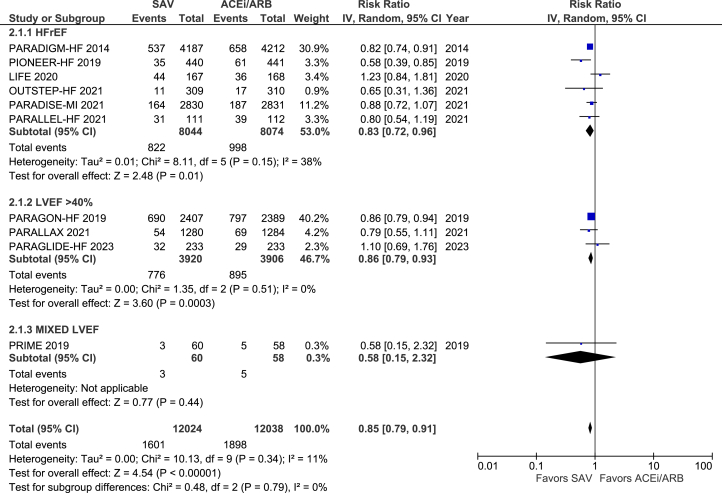
Heart Failure Rehospitalization Abbreviations as in Figure 4.

SAV significantly reduced the rate of HF readmission among the participants irrespective of their baseline LVEF: in EF <40% (RR: 0.83; 95% CI: 0.72-0.96; *P* = 0.01), in EF >40% (RR: 0.86; 95% CI: 0.79-0.93; *P* = 0.0003). HF readmission was significantly lower in the SAV-treated group than in the ACEI/ARB-treated group (RR: 0.85; 95% CI: 0.79-0.91; *P* = 0.00001) (Figure 6).

### Safety endpoint analysis

**Figure 7:**
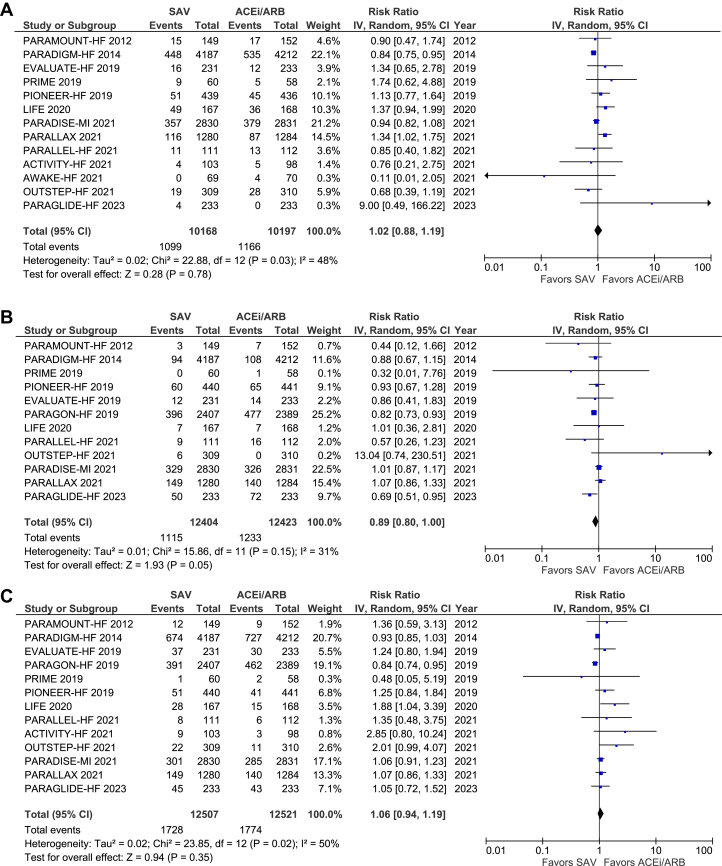

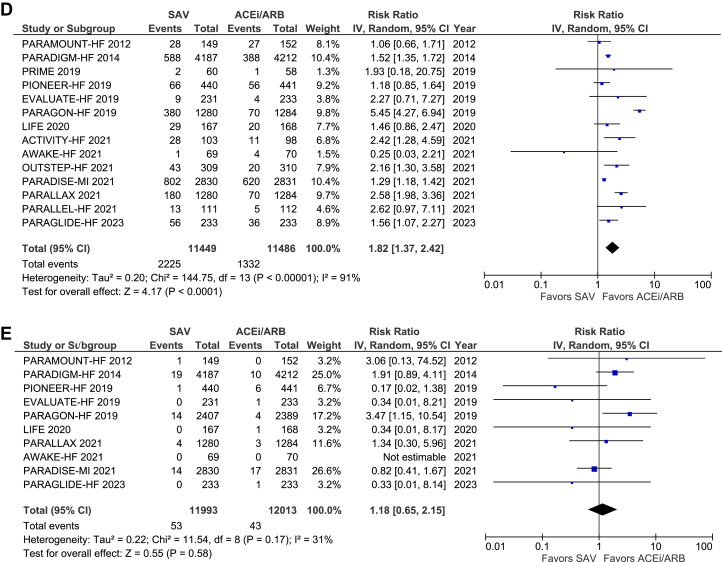
Drug Discontinuation Due to an Adverse Effect, Worsening Renal Function, Hyperkalemia, Hypotension, and Angioedema (A) Drug discontinuation due to an adverse effect. (B) Worsening renal function. (C) Hyperkalemia. (D) Hypotension. (E) Angioedema. Abbreviations as in Figure 4.

Safety profiles between both medications were comparable, except that hypotension occurred more in the SAV-treated group compared to ACEI/ARB (RR: 1.82; 95% CI: 1.37-2.42; *P* < 0.0001) (Figure 7D).

## Discussion

The results from our meta-analysis include: 1) a reduction in all-cause mortality in the combined HF population; 2) subgroup analysis revealed a reduction in all-cause mortality exclusively in the population with EF <40%; 3) no significant difference in CV-related deaths was observed; 4) SAV significantly reduced HF readmission rates across all baseline EFs; and 5) comparative safety analysis between SAV and ACEI/ARB indicated general similarity, barring an increased incidence of hypotension in the SAV-treated cohort (Central Illustration).

**Central Illustration:**
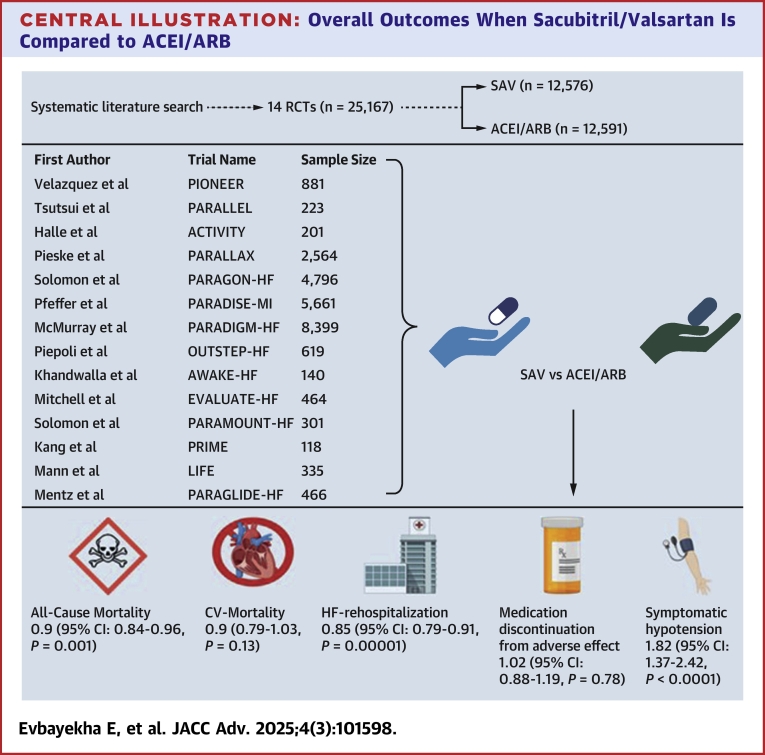
Overall Outcomes When Sacubitril/Valsartan Is Compared to ACEI/ARB CV = cardiovascular; HF = heart failure; RCT = randomized clinical trial; other abbreviations as in Figure 4.

This meta-analysis highlights the superiority of SAV over ACEI/ARB in reducing all-cause mortality in the group with an EF of <40%. This translates into clinical practice as a strong indication to initiate SAV in newly diagnosed individuals with EF <40%, and in those with a longstanding history of ACEI/ARB use, the need to overcome the inertia of transitioning to SAV. The PARADIGM-HF trial likely drove our result, as it was the only positive trial of 14. However, the studies with relatively larger sample sizes (PARADISE-MI [Prospective ARNI vs. ACE Inhibitor Trial to Determine Superiority in Reducing Heart Failure Events After Myocardial Infarction], PARAGON-HF [Prospective Comparison of ARNI with ARB Global Outcomes in Heart Failure with Preserved Ejection Fraction]) showed a statistically nonsignificant reduction in all-cause mortality.^6^^,^^7^ Hence, the negative trials suggest they were likely underpowered to detect a difference. Another plausible explanation for this result could be the design of the PARADIGM-HF trial, which excluded patients with a <3 months history of acute coronary event, only included 5.1% of Black participants, and excluded patients with severe pulmonary disease.^5^^,^^27^ It is not completely understood how SAV confers mortality benefits in HFrEF. Perhaps this stems from the pathophysiology of HF, which, while not mutually exclusive, may differ at some molecular level between those with EF <40% and >40%, which enables SAV to confer benefit.^1^^,^^28^ As noted in existing studies, as EF increases in HF, other extracardiac factors or comorbidities become more impactful in mortality outcomes. Clinically, EF should be treated as a continuum except within the 50% to 55% range.^1^

In contrast to its efficacy in reducing all-cause mortality, SAV did not demonstrate superiority over ACEI/ARB in preventing CV-related deaths across the EF spectrum. This was not unprecedented, as only the PARADIGM-HF trial, among the 8 trials considered in the final analysis, reported a reduction in CV mortality. These findings are consistent with some observational European studies that found no CV mortality benefit of SAV over ACEI/ARBs.^29^ The reason for this is unknown and warrants further studies. Our analysis suggests a nonstatistically significant reduction in CV mortality across the EF spectrum, suggesting that SAV may potentially improve CV mortality in a selected group of HF population. Additionally, the use of optimal background HF therapy, including beta-blockers, mineralocorticoid receptor antagonists, sodium-glucose transporter-2 inhibitors, and diuretics, may account for an overall low CV-related mortality event rate in the SAV and ACEI/ARBs groups.^29^^,^^30^ Moreover, the included studies had unequal comparative dosages of SAV vs ACEI/ARBs in addition to a wide variation in patient follow-up durations (from 27 months in the PARADIGM-HF trial to 8 weeks in the AWAKE-HF [Sacubitril/valsartan Impact on daily physical activity and sleep in heart failure] trial).^5^^,^^31^^,^^30^ These findings buttress the importance of considering the spectrum of EF as a significant determinant of response to HF therapy.

One of the most important findings of this study is the impact of SAV on hospitalization reduction across the EF spectrum. Several studies have demonstrated the efficacy of SAV in reducing hospitalizations compared to ACEI/ARBs. A pooled individual participant-level analysis of the PARADIGM-HF and PARAGON-HF trials revealed that, over a 2.8-year median follow-up duration in >13,000 participants, SAV reduced the risk of hospitalization with an absolute risk reduction of 2.1 per 100 patient-years and a corresponding number needed to treat of 48 patient-years.^32^ Although not completely understood, this reduction in hospitalization in the SAV group may be attributed to reductions in blood pressure and/or diuretic dosage, improvement in left ventricular function, and pulmonary hypertension.^5^^,^^19^^,^^33^ Frequent hospitalizations are associated with worse prognosis and increased mortality in HF patients. By reducing hospitalizations, SAV improves patients' quality of life and alleviates the economic burden on the health care system, making it a valuable treatment option in HF management.

The safety profile analysis confirmed a notable increase in symptomatic hypotension in the SAV group. The plausible mechanism behind this finding can be elucidated through SAV's dual action as a neprilysin inhibitor and ARB. Neprilysin inhibition increases levels of natriuretic peptides, resulting in vasodilation and decreased blood pressure. Simultaneously, the angiotensin receptor blockade can amplify this effect by reducing vasoconstriction. These combined actions may contribute to the observed higher incidence of hypotension in individuals receiving SAV.^28^^,^^30^ It is important to note the moderate to substantial between-study heterogeneity in the safety profile analysis outcomes. This is likely due to the lack of uniformity in trial safety endpoints. Patient-specific factors like age, baseline blood pressure, and comorbidities may influence susceptibility to hypotensive events. The significance of this finding should be considered in the context of the overall CV benefits provided by SAV.^1^ Furthermore, it is important to realize that despite hypotension, both groups had no difference in overall medication discontinuation.

### Strengths and limitations

This study, compared to prior similar publications (Haseeb et al,^34^ Hernandez et al,^35^ among others), is the largest meta-analysis to date that evaluated hard endpoints from 14 randomized clinical trials, with a sample size of n = 25,167, across the spectrum of EFs. The analysis showed very low to absent heterogeneity. These results allow us to confidently make clinical arguments about overcoming inertia when initiating and transitioning patients to SAV. This systematic review and meta-analysis, while comprehensive, has several limitations. Firstly, not all endpoints were consistently available or reported across the included trials, which may affect the robustness of the findings. As a study-level meta-analysis, this study did not have patient-level data to stratify analysis outcomes based on comparative dosages of SAV vs ACEI/ARB. The heterogeneity in safety profile outcomes, particularly regarding hypotension, suggests variability in medication dose titration and patient populations, which could influence the results. The study's reliance on existing trials means that future research could alter the current conclusions. Furthermore, the analysis based on EF may not fully account for the complex interplay of patient-specific factors such as age, comorbidities, and baseline medications, which can impact outcomes. The lack of uniformity in trial safety endpoints and the potential underpowering of some trials to detect differences in CV-specific mortality also limit the generalizability of the findings. Despite these limitations, the study provides valuable insights into the efficacy and safety of SAV compared to ACEI/ARBs in HF management.

## Conclusions

This analysis showed that SAV significantly reduced rehospitalization rates across the EF spectrum with the additional benefit of reducing all-cause mortality in subgroups with an EF of <40%. Thus, clinicians should consider initiating or escalating to SAV for HF management. Monitoring hypotension and tailoring treatment based on the EF spectrum is crucial to ensure optimal outcomes.

## Funding support and author disclosures

The authors have reported that they have no relationships relevant to the contents of this paper to disclose. This study received no funding.
