# Supplementary material for: Sacubitril/Valsartan vs ACE Inhibitors or ARBs: A Systematic Review and Meta-Analysis of Randomized Trials
Source: JACC Adv. 2025 Feb 18;4(3):101598. doi: 10.1016/j.jacadv.2025.101598 (PMC11883387; doi:10.1016/j.jacadv.2025.101598)
Supplement: Supplementary data [file mmc1.docx]

Supplemental Figure 1


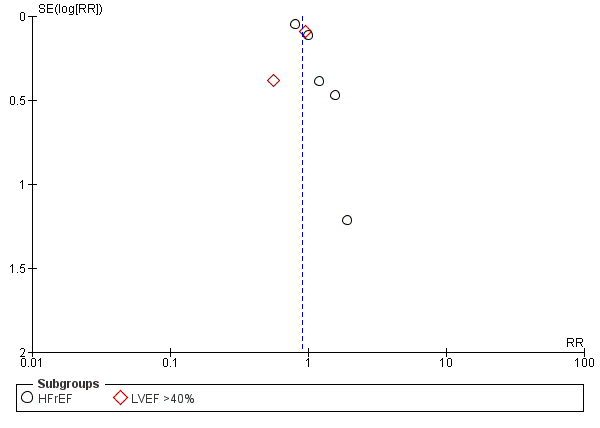


Supplemental Figure 2


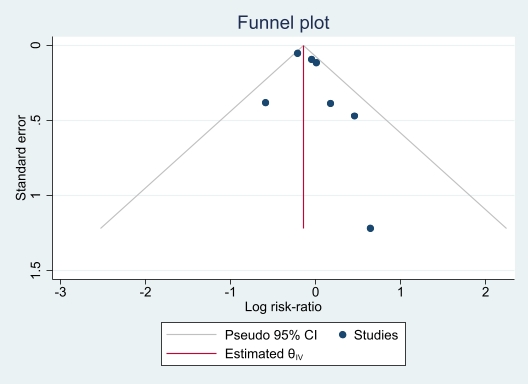


CV mortality funnel plot (Egger’s test for a regression intercept gave a p-value of 0.74, indicating no evidence of publication bias)

Supplemental Figure 3


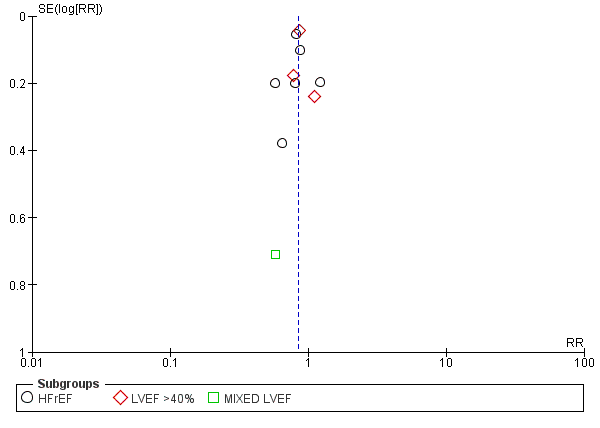


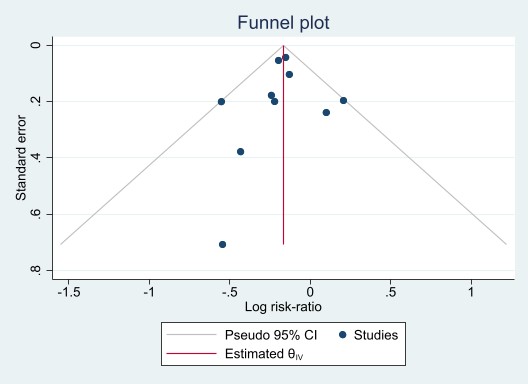


HF Re-admit funnel plot (Egger’s test for a regression intercept gave a p-value of 0.74, indicating no evidence of publication bias)
